# Editorial: Stem Cell-Derived Exosome Therapy of Microbial Diseases: From Bench to Bed

**DOI:** 10.3389/fmicb.2021.842773

**Published:** 2022-01-18

**Authors:** Amin Tamadon, Nader Tanideh, Reza Shirazi

**Affiliations:** ^1^PerciaVista R&D Co., Shiraz, Iran; ^2^Stem Cells Technology Research Center, Shiraz University of Medical Sciences, Shiraz, Iran; ^3^Department of Pharmacology, Medical School, Shiraz University of Medical Sciences, Shiraz, Iran; ^4^Department of Anatomy, School of Medical Sciences, Medicine and Health, University of New South Wales, Sydney, NSW, Australia

**Keywords:** stem cell, exosome (EXO), microbial diseases, therapy, extracellular vesicles

Exosomes are nano-sized vesicles that play a mediator role in cell-to-cell communication. They are composed of unique proteins, lipids and nucleic acids, which replicate the composition of producer cells and can be used as cell-free therapeutics. Exosomes derived from stem cells have attracted great attention due to their immunomodulatory, regenerative and antimicrobial capabilities. These characteristics have been demonstrated in various *in vitro* and *in vivo* models. Furthermore, recent developments in the field of exosome therapy have resulted in elaboration of specific quality control methods and guidelines, which will facilitate the use of exosomes in clinical settings.

The leading cause of death in intensive care units (ICUs) is sepsis, with a mortality rate as high as 25% in severe cases (Fleischmann-Struzek et al., 2020). Microbial infections, which cause sepsis, involve complex interactions between microbial pathogens and the host immune system. Excessive induction of endogenous pro-inflammatory cytokines and coagulation pathways during the early phase of sepsis result in adverse effects in patients. Stem cells can modulate the expression of the corresponding genes in sepsis (Huang et al., 2017). Stem cells also enhance the clearance of pathogens and repair of injured tissues in sepsis. There are new insights for treatment of microbial disease using stem cell-derived exosomes which have been discussed in this Research Topic.

The researchers who contributed to this Research Topic presented 10 themed articles that highlighted the knowledge from recent advancements in the field of exosome therapy of microbial infections. For example, in the work by You et al., we learnt that the various mechanisms of stem cells-derived extracellular vesicles (MSC-EVs) treatment for infectious diseases in detail. The authors described MSC-EVs mechanisms for treatment of intestinal infections, sepsis, and respiratory infections. The authors also demonstrated challenges for implementing MSC-EVs from bench to bedside. In addition, Keshavarz Alikhani et al. verified biogenesis and the fate of EVs. They demonstrated EV-based therapy and current developments in understanding the potential application of stem cell-derived EVs on pathogenic microorganisms. They also highlighted the mechanisms by which EVs were exploited to fight against infectious diseases and the deriver challenges in translation of stem cell-derived EVs into the clinical arena. On the other hand, Keshtkar et al. described that most published studies on stem cell derived-exosomes are preclinical and are under way to reach clinical applications. They emphasized the challenges ahead of this cell-free therapeutic method that might be applied as a treatment alternative to stem cells. By the way, Wu et al. highlighted the latest progress in the clinical translation of the MSCs-derived exosomes therapy, by summarizing related clinical trials, routes of administration and exosome modifications.

Four articles focused on the treatment of specific infectious diseases using MSCs-derived exosomes. Izadi et al. evaluated the studies about the potential therapeutic roles of MSCs-derived exosomes on sperm abnormalities and male infertility caused by sexually transmitted diseases (STDs) including Chlamydia infection. These investigators described that exosomes have potential properties for preventing the consequences of infection, such as reducing cell damage, preventing inflammation, and reducing scar formation by inhibiting fibrogenesis. The second review article by Zohrabi et al. discussed how MSCs-derived exosomes secrete different bioactive factors. These secretions can prevent infection and modulate the immune system. Thus, they reviewed the possible application of MSCs-derived exosomes in female reproductive system bacterial diseases. Furthermore, the other review by Raghav et al. summarized recent findings on the application of the cargo-loaded stem cell-derived exosomes in the treatment of diabetic foot ulcers (DFUs). They also categorized the different approaches for loading the desired cargo/drug inside exosomes. On the other hand, Jafari et al. described the ability of MSCs-derived exosomes as a therapeutic choice for controlling and treatment of orodental infectious diseases.

Considering to fungal disease, Ghasemian discussed applications of stem cell derived exosomes in in fungal diseases. In her review, the probable role of exosomes, limitations for clinical studies and mechanisms of action of exosomes in treating fungal diseases was explored. In a novel insight into exosome therapy of infectious diseases, Bayat et al. presented the first description that algae derived stem cells can produce EVs. They described properties of EVs extracted from this marine derived source and their antimicrobial effects.

In conclusion, this themed collection enhances our knowledge of exosome isolation methods from stem cell for anti-bacterial, antifungal, antiviral, or anti-parasitic applications. The papers particularly highlighted potential targets and methods for stem cell genome manipulation for improved production of antimicrobial agents and release-through exosomes and also summarized *in vitro* and *in vivo* studies evaluating stem cell-derived exosomes on pathogenic microbes. Nevertheless, introducing quality control measures and guidelines for production of stem cell-derived exosomes as antimicrobials in clinical settings needs further research and development.

## Author Contributions

AT drafted the Editorial while NT and RS contributed to editing. All authors conceived and designed the work and provided final approval of the version to be published.

## Conflict of Interest

AT was employed by PerciaVista R&D Co. The remaining authors declare that the research was conducted in the absence of any commercial or financial relationships that could be construed as a potential conflict of interest.

## Publisher's Note

All claims expressed in this article are solely those of the authors and do not necessarily represent those of their affiliated organizations, or those of the publisher, the editors and the reviewers. Any product that may be evaluated in this article, or claim that may be made by its manufacturer, is not guaranteed or endorsed by the publisher.
